# The impact of supervised and home exercise activity intervention on circulating immune cell numbers in cancer patients

**DOI:** 10.1016/j.heliyon.2024.e39320

**Published:** 2024-10-12

**Authors:** Rihacek Michal, Wagnerova Andrea, Halamkova Jana, Pehalova Lucie, Rihackova Eva, Boucek Lubos, Kapounková Kateřina, Hrnčiříková Iva, Kiss Igor

**Affiliations:** a Department of Hematology and Transfusion, AGEL Central Moravia Hospitals, AGEL Prostejov Hospital, Mathonova 1, 796 01, Prostejov, Czechia; b Department of Laboratory Medicine, AGEL Central Moravia Hospitals, Mathonova 1, 796 01, Prostejov, Czechia; c Department of Laboratory Methods, Faculty of Medicine, Masaryk University, Kamenice 5, 625 00, Brno, Czechia; d Department of Laboratory Medicine, University Hospital Brno, Jihlavska 20, 625 00, Brno, Czechia; e Department of Comprehensive Cancer Care, Masaryk Memorial Cancer Institute, Zluty Kopec 7, 656 53, Brno, Czechia; f Institute of Biostatistics and Analyses, Faculty of Medicine, Masaryk University, Kamenice 126/3, 62500, Brno, Czechia; g Institute of Health Information and Statistics of the Czech Republic, Palackeho namesti 4, 12801 Prague 2, Czechia; h Department of Internal Medicine and Cardiology, University Hospital Brno and Faculty of Medicine, Masaryk University, Jihlavska 20, 625 00, Brno, Czechia; i Department of Physical Activities and Health Sciences, Faculty of Sports, Masaryk University, Kamenice 5, 625 00, Brno, Czechia

**Keywords:** Exercise, Immune cells, Oncology, Fitness, Chemotherapy

## Abstract

**Introduction:**

Malignant diseases challenge clinicians to balance treatment intensity and patient quality of life. Regular physical activity positively impacts mental and physical health, benefiting sleep patterns, heart rate, and overall health. Moreover, telehealth physical exercise training represents a viable option for maintaining intrinsic capacity. The American Cancer Society highlights exercise's role in helping patients cope with anti-cancer treatment side effects. In the Czech Republic, there is no fitness-promoting protocol for cancer patients, despite recognized benefits. Exercise may also enhance immune function, with moderate-intensity exercise potentially positively affecting immune cell counts.

**Objective:**

This study aimed to analyze the long-term effects of exercise on circulating immune cells in patients undergoing treatment for solid malignancies.

**Patients and methods:**

49 participants were recruited at the Masaryk Memorial Cancer Institute, Czech Republic, starting September 2021. Participants were randomized into an experimental (SAPA = undergoing monitored exercise program) group (N = 16; madian age: 46,6; median BMI: 25,1) and a control (CO) group (N = 33; madian age: 52,0; median BMI: 25,3). Flow cytometry was used to examine cellular immunological profiles. The exercise program involved thrice-weekly sessions conducted online.

**Results:**

SAPA group showed stable lymphocyte counts post-exercise (percentage chance: +0,5 %; p = 0,256; effect size r = −0,284), while the CO group exhibited a significant drop (percentage chance: −23,0 %; p = 0,015; effect size r = −0,423). B lymphocyte numbers were significantly higher in the SAPA group post-exercise compared to the CO group (p = 0,003; effect size r = −0,422). The number of Th-lymphocytes, T-c lymphocytes, T-gamma/delta lymphocytes, and NK cells remained stable in SAPA but dropped in CO group.

**Conclusion:**

Exercise's impact on the immune system in cancer patients shows promise, with differences noted between acute and chronic exercise effects. Previous studies on acute exercise indicate a rise in immune cell counts, supporting our findings of stable or increased immune cells with controlled exercise in cancer patients. Controlled physical activity stabilizes or increases certain immune cell populations in patients undergoing chemotherapy for solid malignancies, highlighting the potential benefits of incorporating exercise into cancer treatment protocols.

## 1 Introduction

Malignant diseases pose a challenge for clinicians in terms of balancing treatment intensity and maintaining quality of life during and after treatment. Regular physical activity positively affects mental and physical health [1]. Daily moderate-intensity exercise was reported to have a beneficial effect on sleep pattern, heart rate, and overall health status [2].

Among the comprehensive guidelines for non-pharmacological therapy for cancer patients are those issued by the American Cancer Society (ACS). In 2008, the ACS published detailed recommendations for patients undergoing cancer treatment. The introduction of regular physical activity for cancer survivors after chemotherapy is recommended not only by the American College of Sports Medicine (ACSM) in its 2018 guidelines but also by WHO and AICR (American Institute for Cancer Research) and WCRF (World Cancer Research Fund). All these institutions emphasize controlled physical activity (PA) for patients and the combination of aerobic and resistance training. These guidelines consider regular physical activity for cancer patients to be safe and beneficial on multiple levels, particularly in terms of quality of life. However, it is essential to tailor physical activity to the individual needs and health status of patients [[3], [4], [5]]. According to the ACSM, there is strong evidence supporting the popularity of exercise oncology in the exercise and medical communities either using virtual or in-person training [6]. Additionally, combined aerobic and resistance training appears to be the most effective and feasible exercise approach for people with cancer [7,8].

Exercise does not only benefit patients in maintaining their fitness, but also help them cope with anti-cancer treatment and its side effects. Physical exercise should be encouraged by clinical oncologists in their outpatient offices as a part of monitoring overall quality of life, however determining type, intensity and duration of selected activities is difficult and requires an individualized approach [9]. In the Czech Republic, there is currently no fitness-promoting treatment protocol for patients with malignancies, that would define individualized physical activities during anti-cancer treatment, besides general recognition of its possible profit.

Apart from benefits on physical condition, quality of life, weight management, cardiovascular health and mental health [9], some studies suggest that regular, moderate-intensity exercise may positively impact immune function [10]. Regarding immune cell counts in blood circulation, high-intensity training showed immediate rise in white blood cell counts followed by its drop back to baseline. In contrast low intensity does not seem to have significant impact on leukocyte counts [11,12]. More in-depth analysis of cell subsets during exercise-induced leukocytosis in cancer patients showed 10-fold rise in natural killer (NK) cell and 2,5-fold rise in CD8^+^ T cells numbers in 45–60 min, that is believed to be caused by stimulation of beta-2-adrenergic receptors on these cells [13]. This phenomenon is further supported by higher presence of beta-2-adrenergic receptors on NK-cells and CD8^+^ T cells than on B cells and CD4^+^ T-cells [13]. Immune cell response to malignant cell is of a great importance during anti-cancer treatment. It has been postulated, that exercise may lower tumor hypoxia by various mechanisms such as vasodilatation and higher perfusion of tumor tissue. This leads to increased turnover of immune cells in tumor tissue, resulting in better immune response [13].

Our goal was an in-depth analysis of cell populations by flow cytometry to elucidate possible long-term effects of exercise on the number of circulating cells in patients treated for solid malignancies.

### 1.1 Patients and methods

**Fig. 1:**
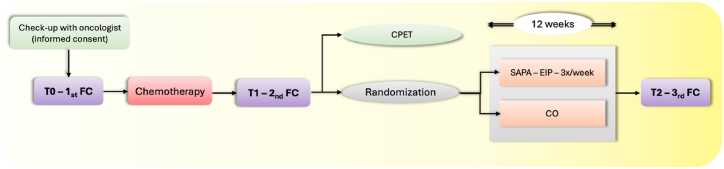
Detail of study design concerning enrollment into the study, randomization and flow cytometry in control (CO) group and exercise (SAPA) group. FC = flow cytometry, SAPA = experimental group (supervised physical activity), CO = control group, CPET = cardiopulmonary exercise testing, EIP = exercise intervention program.

A total of 49 participants were enrolled in the study (detail in Table 1), study design concerning enrollment into the study and randomization is mentioned in Fig. 1. The recruitment of subjects began in September 2021. All patients were treated and recruited at the Masaryk Memorial Cancer Institute (Brno, Czech Republic). The patients were approached by an oncologist with a proposal to participate in the project in the form of a signed worksheet and written informed consent which was requested by all participants. Subsequently, participants were introduced to the project. Inclusion criteria were set as follows: histologically confirmed diagnosis of malignant tumor with indicated adjuvant chemotherapy based on platinum compounds, taxanes or vinca alkaloids (1), Easter Cooperative Group (ECOG) performance status 0, 1 or 2 (2), the ability to walk 400m without sitting or the help of another person (3) and an estimated survival time of at least 9 months (4). Exclusion criteria were set as follows: inability to perform physical activity (1), terminal stage of an oncological or other disease (2), untreated or uncontrolled disease of the lungs, joints or cardiovascular system (3), prior cardiovascular event (i.e. stroke, myocardial infarction) within past 6 moths (4), acute or chronic disease of the immune system or other disease that directly affects this system (e.g. lupus erythematosus, rheumatoid arthritis, immunosuppressive treatment) (5), pregnancy or breastfeeding (6), current treatment with beta-blockers (7), VO2max as an athlete (8). Participants were divided into groups due to computer-generated randomization. Randomization occurred in the order of participant completion of baseline testing with study staff kept blinded to group allocation until the baseline assessment was complete.

**Table 1:** The basic characteristics of an enrolled cohort of patients.

	Experimental (SAPA) group (N = 16)	Control (CO) group (N = 33)	p-value [1]
**Sex**			
Female	15 (93,8 %)	28 (84,8 %)	0,649
Male	1 (6,3 %)	5 (15,2 %)	
**Age**			
0–50	10 (62,5 %)	15 (45,5 %)	0,445
51–64	4 (25,0 %)	9 (27,3 %)	
<65	2 (12,5 %)	9 (27,3 %)	
Average (SD)	49,2 (10,4)	53,8 (14,2)	
Median (25%–75 % percentile)	46,6 (41,5–55,1)	52,0 (42,1–65,4)	0,228
**BMI**			
18,5–24,9	8 (50,0 %)	16 (48,5 %)	0,873
25–29,9	6 (37,5 %)	11 (33,3 %)	
30 a více	2 (12,5 %)	6 (18,2 %)	
Average (SD)	25,7 (3,6)	25,9 (4,7)	
Median (25%–75 % percentile)	25,1 (22,3–28,1)	25,3 (22,0–28,9)	0,839
**Diagnosis**			
Breast cancer	15 (93,8 %)	28 (84,8 %)	0,649
Other	1 (6,3 %)	5 (15,2 %)	
**Clinical stage**			
Stage I	5 (31,3 %)	12 (36,4 %)	0,900
Stage II	9 (56,3 %)	18 (54,5 %)	
Stage III	2 (12,5 %)	3 (9,1 %)	
**Type of the Treatment**			
S + CH + R	6 (37,5 %)	10 (30,3 %)	0,895
S + CH + R + H	9 (56,3 %)	20 (60,6 %)	
S + CH	1 (6,3 %)	3 (9,1 %)	
**Treatment duration**			
1–4 months	6 (37,5 %)	16 (48,5 %)	0,549
5–6 months	10 (62,5 %)	17 (51,5 %)	
**Chemotherapy type**			
Cisplatin	1 (6,3 %)	2 (6,1 %)	0,811
Oxaliplatin	0 (0,0 %)	3 (9,1 %)	
Docetaxel	1 (6,3 %)	1 (3,0 %)	
Paclitaxel	14 (87,5 %)	27 (81,8 %)	

Shortcuts: S = surgery, CH = chemotherapy, R = radiotherapy, H = hormone therapy.

Across all subjects, flow cytometric analysis of cellular immunological profiles was conducted at predetermined intervals: prior to the commencement of chemotherapy (T0) and following its completion (T1). Upon completion of chemotherapy patients were randomized into two groups: the SAPA group, which underwent a 12-week online supervised physical activity regimen, and the control group, which continued standard treatment without additional intervention. Cardiopulmonary exercise testing (CPET) was administered to determine the appropriate intensity of exercise for each participant. Concurrently, blood samples were collected for flow cytometric analysis at T2 (following the 12-week intervention period) from subjects in both groups (as illustrated in Fig. 1). This methodology allowed for a comprehensive assessment of the potential immunomodulatory effects of structured physical activity in post-chemotherapy patients.

### 1.2 Exercise intervention program

„The exercise intervention program was started no later than 6 weeks after the completion of chemotherapy and lasted for 12 weeks, with sessions held 3 times a week for 60 min each. All sessions were conducted online via the ZOOM platform, and the exercises were supervised by instructors. The warm-up, lasting approximately 10 min, included dynamic stretching of the whole body and mobilization exercises. The cooldown, also about 10 min, involved static stretching, calming breathing exercises, and relaxation. The main part of the session focused on aerobic interval training, consisting of 45 s of exercise followed by 15 s of rest, with alternating exercises, and lasted about 45 min. The intensity of physical exertion was monitored using sport testers and subjective perception of effort (Borg scale). The intensity of the exercise was tailored for each participant based on initial load testing, with heart rate maintained within the prescribed range of 60–80 % of maximum heart rate reserve, or VO2max. After each exercise session, participants were asked to report their maximum and average heart rate.“

### 1.3 Flow cytometry assay

For the study of immune system cells, flow cytometry was employed as a laboratory method enabling rapid multiparametric analysis. In a cellular suspension, the immunophenotype and cell quantity were determined using fluorochrome-labeled monoclonal antibodies. For these purposes, peripheral blood (with K_3_EDTA anticoagulant) was collected from patients at specified intervals. Each patient's peripheral blood sample was analyzed in 3 panels with the following combination of CD markers and fluorochromes (see Table 2).

**Table 2:** Overview of used fluorochrome-conjugated antibodies (Beckman Coulter).

Panel 1		Panel 2		Panel 3	
Marker	Fluorochrome	Marker	Fluorochrome	Marker	Fluorochrome
CD45	KrO	CD45	Kro	CD4	PB
CD19	PC7	CD14	PE	CD25	PC5.5
CD3	AF750	CD33	FITC	CD127	PE
CD4	PB	CD16	PC7		
CD8	APC	CD11b	APC		
CD14	PE	CD15	PB		
CD16	PE	HLA-Dr	PC5.5		
CD56	PE				
TCR γ/δ	FITC				

**Fig. 2:**
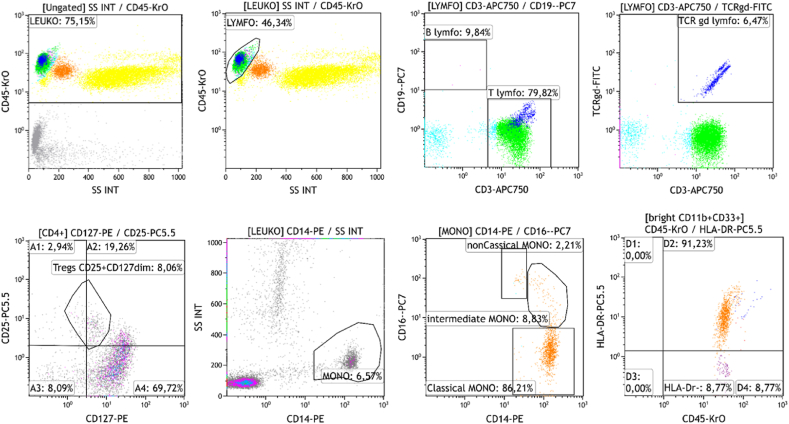
Overview of used fluorochrome-conjugated antibodies (Beckman Coulter) Gating strategy: Leukocyte count is gated by CD45-KrO antibody and side scatter parameters (SSC). Lymphocyte protocol: gated as CD45^+^ cells with low side scatter. B-cells are CD3^−^CD19^+^ in the next scattergram and T-cells as CD3^+^CD19^−^. Tcr-gd subsets are CD3^+^ and TCRgd-positive in the next scattergram. In a following scattergram T-regulatory lymphocytes are identified using CD25-PC5.5 antibody and CD127-PE antibody as CD25^+^CD127dim. Monocyte protocol: gated as CD14^+^ with low SSC. Monocytic subpopulations are subdivided using CD16-PC7 antibody and CD14-PE antibody to classical monocytes CD14++CD16^−^, intermediate monocytes CD14++CD16^+^ and non-classical monocytes CD14^+^CD16++.

Sample preparation for flowcytometric analysis included the addition of an antibody mixture to patient sample in a polypropylene tube, followed by the addition of peripheral blood at a volume of 50 μl. Subsequently, incubation for 15 min in the dark at room temperature was performed. In the second step, VersaLyse Lysing Solution was used for erythrocyte lysis during a 15-min incubation in the dark at room temperature. Finally, unbound antibodies were washed away using PBS buffer (PBS – Phosphate-Buffered Saline, phosphate-buffered physiological solution; Beckman Coulter) and sample was analyzed on Beckmann Coulter Navios Ex flowcytometer – see gating strategy (Fig. 2).

### 1.4 Statistical analysis

Comparisons of baseline patient characteristics between the SAPA and CO groups were summarized with counts and frequencies and tested by Fisher's exact test in case of categorial characteristics. To determine the effect size, we calculated the r coefficient. Continuous characteristics were described using the median and the 25th-75th percentile and tested with the Mann-Whitney *U* test. The impact of chemotherapy on blood parameters was assessed using the median and the 25th-75th percentile before (T0) and after (T1) chemotherapy, and the Wilcoxon test was applied. The 12-week exercise intervention program's effect on blood parameters was described using the median and the 25th-75th percentile before and after the program in the SAPA group (and in the CO group for the corresponding time period). Changes in blood parameters over the specified time period for the SAPA and CO groups were evaluated with the Wilcoxon test. Comparisons of parameters changes between the groups were made using the Mann-Whitney *U* test.

## 2 Results

1. Absolute cell counts and chemotherapy.

Changes in observed parameters were initially evaluated with regards to administration of chemotherapy (Table 3.) in defined interval T0 (prior chemotherapy) and T1 (after chemotherapy). As expected, significant drop in red blood cell counts (percentage chance: −9,2 %; p < 0,001; effect size r = −0,757) and leukocyte counts (percentage chance: −19,5 %; p < 0,001; effect size r = −0,644) acquired by CBC automated analysis was observed. The drop of neutrophil (percentage chance: −18,7 %; p < 0,001; effect size r = −0,536) and lymphocyte (percentage chance: −31,3 %; p < 0,001; effect size r = −0,623) populations was the main cause of the decrease in leukocytes. Immunophenotyping of lymphocyte subpopulations showed underlying statistically significant drop of B-lymphocytes (percentage chance: −63,9 %; p < 0,001; effect size r = −0,777) and NK-cells (percentage chance: −15,4 %; p = 0,019; effect size r = −0,334). Moreover, chemotherapy negatively influenced T-regulatory lymphocyte numbers (percentage chance: −28,6 %; p < 0,001; effect size r = −0,643) (see Table 3.) (see Table 4).

2. Absolute cell counts and effect of exercise.

**Table 3:** Absolute cell counts and relation to chemotherapy according to subgroups (N = 49).

Selected parameter (abs. count∗10^9^/l) Median (25%–75 % percentile)	SAPA group (N = 16)			Control group (N = 33)			SAPA vs. Control group
	Prior to chemotherapy (T0)	After chemotherapy (T1)	p-value^a^	Prior to chemotherapy (T0)	After chemotherapy (T1)	p-value^a^	p-value^b^
Erytrocytes	4,35 (4,07–4,50)	3,91 (3,58–4,15)	**0,004**	4,45 (4,15–4,75)	4,03 (3,71–4,39)	**< 0,001**	0,790
Leukocytes	6,41 (5,71–8,70)	5,53 (4,04–6,23)	0,005	6,74 (5,46–8,07)	5,23 (4,38–6,47)	<0,001	0,966
Hemoglobin	128,0 (120,3–136,8)	120,0 (112,3–126,3)	**0,008**	132,0 (124,0–136,5)	122,0 (116,5–129,5)	**< 0,001**	0,983
Trombocytes	256,5 (245,3–316,8)	279,0 (243,0–335,0)	0,691	258,0 (230,0–284,5)	256,0 (213,0–325,0)	0,260	0,815
Neutrophils	3,62 (3,13–5,71)	2,97 (2,51–4,29)	**0,044**	4,21 (3,25–4,97)	3,22 (2,52–4,37)	**0,002**	0,709
Eosinophils	0,10 (0,05–0,14)	0,11 (0,05–0,20)	0,637	0,09 (0,05–0,20)	0,12 (0,08–0,22)	0,557	0,798
Basophils	0,050 (0,040–0,060)	0,045 (0,033–0,050)	**0,016**	0,040 (0,025–0,050)	0,040 (0,030–0,060)	0,371	**0,030**
Monocytes	0,49 (0,35–0,57)	0,54 (0,34–0,66)	0,605	0,47 (0,38–0,59)	0,49 (0,37–0,65)	0,758	0,814
Lymphocytes	2,11 (1,45–2,30)	1,09 (1,01–1,48)	**0,002**	1,94 (1,53–2,37)	1,53 (1,06–1,81)	**0,002**	0,232
B-lymphocytes	0,18 (0,10–0,24)	0,03 (0,01–0,05)	**< 0,001**	0,03 (0,02–0,04)	0,01 (0,00–0,03)	**< 0,001**	**< 0,001**
NK-cells	0,26 (0,20–0,38)	0,22 (0,18–0,28)	0,103	0,22 (0,17–0,36)	0,19 (0,16–0,30)	0,104	0,749
NK-T cells	0,12 (0,09–0,20)	0,13 (0,06–0,20)	0,438	0,12 (0,07–0,19)	0,14 (0,09–0,22)	0,242	0,205
T gama/delta cells	0,034 (0,020–0,111)	0,031 (0,015–0,108)	0,277	0,034 (0,023–0,068)	0,032 (0,023–0,059)	0,888	0,321
CD14^+^CD16^−^ monocytes	0,42 (0,34–0,49)	0,46 (0,28–0,53)	0,836	0,41 (0,32–0,53)	0,41 (0,31–0,51)	0,837	0,974
CD14^+^CD16++ monocytes	0,033 (0,022–0,038)	0,043 (0,028–0,061)	0,078	0,029 (0,022–0,046)	0,041 (0,026–0,059)	0,386	0,212
CD14^+^CD16^+^ monocytes	0,018 (0,013–0,025)	0,023 (0,011–0,029)	0,234	0,014 (0,009–0,027)	0,018 (0,010–0,025)	0,537	0,631
monocyte, MDSC cells	0,004 (0,001–0,007)	0,003 (0,001–0,009)	0,482	0,003 (0,000–0,006)	0,003 (0,001–0,009)	0,905	0,684
granulocyte, MDSC cells	0,10 (0,04–0,13)	0,14 (0,05–0,18)	0,326	0,09 (0,04–0,16)	0,13 (0,08–0,19)	0,138	0,848
T-regulatory lymphocytes	0,15 (0,10–0,16)	0,10 (0,08–0,11)	**0,010**	0,14 (0,10–0,17)	0,10 (0,07–0,13)	**< 0,001**	0,991
Immature granulocytes	0,02 (0,01–0,04)	0,02 (0,01–0,05)	0,647	0,02 (0,01–0,03)	0,02 (0,01–0,03)	0,679	0,819

a Exact p-value for Wilcoxon test.

b Exact p-value for Mann-Whitney *U* test.

**Table 4:** Absolute cell count and its relation to exercise (movement activity).

Selected parameter (abs. count∗10^9^/l) Median (25%–75 % percentile)	SAPA group (N = 16)			Control group (N = 33)			SAPA vs. Control group
	Before excercise (T1)	After excercise (T2)	p-value^a^	T1	T2	p-value^a^	p-value^b^
Erythrocytes	3,91 (3,58–4,15)	4,32 (4,10–4,61)	**0,001**	4,03 (3,71–4,39)	4,47 (4,10–4,76)	**< 0,001**	0,359
Leukocytes	5,53 (4,04–6,23)	4,63 (4,06–5,49)	0,281	5,23 (4,38–6,47)	4,92 (4,13–6,73)	0,957	0,254
Hemoglobin	120,0 (112,3–126,3)	127,0 (122,3–134,5)	**0,008**	122,0 (116,5–129,5)	128,0 (124,5–136,0)	**< 0,001**	0,669
Thrombocytes	279,0 (243,0–335,0)	249,0 (222,5–266,8)	**0,011**	256,0 (213,0–325,0)	228,0 (198,0–267,0)	**< 0,001**	0,656
Leukocytes – FLOW	5,53 (4,04–6,23)	4,63 (4,06–5,49)	0,281	5,23 (4,38–6,47)	4,92 (4,13–6,73)	0,957	0,254
Monocytes – FLOW	0,54 (0,34–0,66)	0,48 (0,43–0,55)	0,513	0,48 (0,36–0,62)	0,44 (0,39–0,56)	0,543	0,773
Lymphocytes – FLOW	1,09 (1,01–1,48)	1,10 (0,80–1,34)	0,256	1,52 (0,96–1,79)	1,17 (0,83–1,59)	**0,015**	0,873
T-lymphocytes	0,88 (0,73–1,12)	0,82 (0,56–1,10)	0,163	1,16 (0,80–1,43)	1,00 (0,67–1,19)	**0,002**	0,831
T-h lymphocytes	0,56 (0,44–0,72)	0,44 (0,38–0,58)	0,056	0,68 (0,48–0,86)	0,54 (0,41–0,71)	**0,001**	0,848
T-c lymphocytes	0,31 (0,26–0,45)	0,30 (0,24–0,35)	0,313	0,41 (0,30–0,62)	0,43 (0,23–0,52)	**0,041**	0,983
B-lymphocytes	0,03 (0,01–0,05)	0,07 (0,04–0,14)	**0,008**	0,01 (0,00–0,03)	0,02 (0,01–0,03)	**0,017**	**0,003**
NK-cells	0,22 (0,18–0,28)	0,17 (0,11–0,23)	0,205	0,19 (0,16–0,30)	0,18 (0,10–0,27)	**0,030**	0,881
NK-T cells	0,13 (0,06–0,20)	0,14 (0,08–0,26)	0,737	0,14 (0,09–0,22)	0,14 (0,08–0,22)	0,238	0,348
T gama/delta cells	0,031 (0,015–0,108)	0,034 (0,015–0,108)	0,641	0,032 (0,023–0,059)	0,033 (0,018–0,045)	**0,009**	0,240
CD14^+^CD16^−^	0,46 (0,28–0,53)	0,41 (0,34–0,45)	0,679	0,41 (0,31–0,51)	0,38 (0,30–0,50)	0,268	0,782
CD14^+^CD16++	0,04 (0,03–0,06)	0,05 (0,03–0,07)	0,393	0,04 (0,03–0,06)	0,04 (0,03–0,08)	0,054	0,725
CD14^+^CD16^+^	0,02 (0,01–0,03)	0,02 (0,01–0,03)	0,379	0,02 (0,01–0,02)	0,02 (0,01–0,02)	0,448	0,757
monocyte, MDSC	0,003 (0,001–0,009)	0,003 (0,001–0,005)	0,430	0,003 (0,001–0,009)	0,002 (0,001–0,006)	0,218	0,966
granulocyte, MDSC	0,14 (0,05–0,18)	0,12 (0,07–0,23)	0,224	0,13 (0,08–0,19)	0,11 (0,07–0,17)	0,586	0,186
T-regulatory lymphocytes	0,10 (0,08–0,11)	0,10 (0,07–0,10)	0,506	0,10 (0,07–0,13)	0,10 (0,08–0,12)	0,957	0,613
Immature granulocytes	0,02 (0,01–0,05)	0,02 (0,01–0,02)	0,073	0,02 (0,01–0,03)	0,02 (0,01–0,02)	**0,013**	0,401

a Exact p-value for Wilcoxon test.

b Exact p-value for Mann-Whitney *U* test.

Monitoring of main blood cell subsets and hemoglobin showed comparable trends between SAPA and CO groups in routinely monitored parameters, that are part of CBC measurement (e.g. leukocytes, erythrocytes, and platelets). In white blood cell differential obtained by immunophenotyping, a visible trend was observed in absolute numbers of lymphocyte population (table 4). SAPA group had stable lymphocyte counts during 12-week exercise period (percentage chance: +0,5 %; p = 0,256; effect size r = −0,284) whereas in CO group statistically significant drop in lymphocyte counts was discovered (percentage chance: −23,0 %; p = 0,015; effect size r = −0,423). Subsequent lymphocyte subsets phenotyping revealed a statistically significant rise in B lymphocyte numbers in both groups (SAPA: percentage chance: +101,5 %; p = 0,008; effect size r = −0,660; CO group: percentage chance: +88,9 %; p = 0,017; effect size r = −0,416). Moreover the rise in B-cell numbers was significantly higher in SAPA group than in control group (p = 0,003; effect size r = −0,422). Absolute counts of Th-lymphocytes, T-c lymphocytes, T-gamma/delta lymphocytes and NK-cells remained quite stable in SAPA group and presented a statistically significant drop in control group, however when compared, the difference between groups was not statistically significant. The number of NK-T cells remained stable during 12-week period in both groups with no difference when comparing results in both groups. T-regulatory lymphocytes presented with no substantial trend in both groups.

## 3 Discussion

It has been widely recognized, that both acute (high-intensity) and chronic (low-moderate intensity) training impacts overall immune system function in healthy people and in cancer patients and survivors [10,14,15]. This statement is particularly important as it was previously thought that cancer patients should rest after the diagnosis of cancer and during anti-cancer treatment.

The benefit of acute (high-intensity) exercise on the immune system seems to differ from that of low-moderate intensity and chronic exercise. Current literature presents more data on benefit of acute training in cancer patients. Study by Koivula et al. performed on newly diagnosed (previously untreated for cancer) breast cancer patients undergoing 10-min acute exercise revealed a total blood leukocyte increase by 29 %, T cell count increase by 34 % and B cell count increase by 18 % [16]. Moreover, a high rise of NK-cell counts was discovered in this study, which supports results of another study by Evans et al. on breast cancer survivors [17]. These findings are in accordance with other studies performed on healthy population during acute exercise [14,16].

To date, less studies seem to have been conducted on patients with solid malignancies undergoing chronic or low-moderate intensity exercise. Thus, comparing our results to those in the literature is limited. The most recent work comparable to our study setup is a study by Arana Echarri et al. that evaluated immune cell counts in 20 breast cancer survivors after 8 weeks of exercise. Results of this study showed comparable results prior and after the exercise period except for lower counts of CD4^+^ EMRA T cells and their activation. However, this study did not include control group for comparison [18]. This is particularly important to note, because we observed a statistically significant drop of CD4^+^ lymphocytes in our control group (as well as in SAPA group, however there it was not statistically significant). It is also important to note that CD4^+^ numbers did not differ significantly between our SAPA and CO groups and that we did not examine EMRA subsets.

The mechanisms by which physical activity influences B-cell lymphocyte counts are multifaceted. Exercise has been shown to induce the release of various cytokines and growth factors that can stimulate the production and mobilization of B cells from the bone marrow [19,20]. Additionally, increased blood flow during exercise can facilitate the migration of B cells to peripheral tissues, enhancing their availability to mount an effective immune response [19]. Physical activity can mitigate the immunosuppressive effects often observed in cancer patients due to chronic inflammation, stress, and treatment side effects [15,21]. By reducing inflammation and stress hormones like cortisol, exercise may create a more favorable environment for B-cell function and survival [22,23]. In summary, exercise may boost B cell numbers by increasing both mobilization and survival.

There have been some reports, that mobilisation of B cells occurs after acute exercise (as mentioned above) [19,24], however majority of studies found minor [25] or no [26] changes in B cell counts in response to prolonged exercise. Some studies confirmed the effect of exercise on B cell numbers among healthy individuals [20,27]. However, this phenomenon was not observed by Arana Echarri on cancer patients but appeared in our study in both SAPA and CO group of patients who finished the anti-cancer treatment cycle recently. The rate of increase was significantly higher in SAPA group than in CO group suggesting the beneficial effect of movement activity on the regeneration rate of B cell numbers post-chemotherapy. As we demonstrated in our study, the number of B cells decreases during chemotherapy – an observation that was previously published in other studies. Moreover, B-cell numbers seemed to be a prognostic factor and patients with lower B-lymphocyte counts presented with less favorable outcomes in ovarian cancer, breast cancer and sarcoma [[28], [29], [30]]. Therefore, supporting B-cell mediated immune function via various modalities such as immunotherapy but also sustained adequate training activity during chemotherapy may improve treatment outcomes.

The role of B cells in anti-cancer immunity is still less understood than that of T cells. B cells have been shown to play roles in anti-tumor immunity by facilitating the stimulation and clonal expansion of T cells [31,32]. This is particularly important because tumor-infiltrating CD8^+^ T cells are associated with increased survival in various human cancers [33]. This positive effect of B cell on T cell resposes was previously confirmed on murine models [32,34]. It is important to note that there have been reports that B cell may rather induce non-anti-tumour humoral immunity or promote metastasis [35,36]. These conflicting results were previously explained by different activation status of B cells, where resting B cell inhibit T cell responses and activate B cells facilitate these responses [33]. Analysis of tumor microenvironment revealed that tumor-infiltrating B cells cover all developmental stages from naive B cells to plasma cells and that these cells are either infiltrating tumor epithelium or organize in tertiary lymphoid structes [37,38]. These tumor-infiltrating B cells have mostly been studied in breast cancer. It has been revealed that they may comprise up to 40 % ot total tumor-infiltrating lymphocyte (TIL) population [39,40]. A positive effect of B cells on inhibiting tumor proliferation has been shown in node-negative breast cancer and also in medullary breast cancer [41,42]. In summary, the role of B cell in tumorigenesis and anti-tumor immunity together with its mediation is a complex topic that remains to be fully elucidated by future studies.

Other immune cell subsets exhibited no notable diferences between SAPA and CO groups in agreement with the results presented by Arana Echarri et al. It is important to note here, that comparison of studies conducting activity tracking relations to any observed parameter is complicated, as the rate and duration of exercise is not standardized. For future prospects, it is important to promote randomized prospective controlled trials to support our results and to elucidate possible beneficial effect of exercise on immune cell counts but more importantly on cancer patients’ treatment outcomes.

### 3.1 Study strengths and limitations

One of the study strengths from the exercise intervention perspective was, that patients performed exercises 3 times a week in an online exercise class under the supervision of a trainer and an assistant. The practice was live from 8 a.m. to 9 a.m. There were immediate interactions between trainer and patient. Each patient has his heart rate monitor (sporttester) personally adjusted, which gives him a response. The patient was checked by a trainer and an assistant, and exercise corrections were made through the camera. The patients were in a home environment, they did not have to commute from distant places. The exercise was adapted to home conditions. This setup provided an advantage, where the patients are led to learn to exercise in the home environment with the tools they are familiar with. This settings leads to increase robustness of repeating session with time. On the other hand telehealth physical exercise training isolates subjects from interaction and potential formation of social group. Exercising in a group rather than alone results in significantly higher pain thresholds. Additionally, cues that promote social bonding before exercising enhance performance outcomes without leading to an increase in perceived fatigue. Online interaction between enrolled cancer patients was carries less bonding experience between persons. However, it carries an advantage in flexibility, wider access to online resources and possible interaction with national or global community.

As for the laboratory results part, potential limitations of the study include the sample size of only 49 participants which limits study's power to detect significant differences. One of the strengths of this study is the application of statistical methods capable of effectively evaluating skewed data and addressing imbalanced group sizes. However, a significant limitation is the small sample size, particularly within the SAPA group. With a reduced number of observations, the statistical power to identify significant differences or correlations might be compromised. This limitation heightens the risk of Type II errors (the failure to identify true effects) and may influence the overall reliability of the findings. Another weakpoint may be that the patients were recruited from a single institution as this was a pilot study in the Czech Republic. Furthermore, the study was not design to include long-term longitudinal data that would go beyond the 12-week period, however such data might be necessary to better understand and describe immune system changes in enrolled subjects. More broad spectrum of blood cell populations might need to be assessed to better understand obtained results in the future.

### 3.2 Future perspectives and clinical implications

Physical activity (PA) has become an increasingly recognized component of cancer care, especially in the post-treatment phase. The evidence supports its role in improving the quality of life, reducing recurrence risks, and enhancing overall survival among cancer survivors. Future research is likely to focus on personalized exercise prescriptions tailored to individual cancer types, treatment side effects, and patient preferences. Future cancer treatment models may integrate physical activity as a core component, emphasizing its role alongside nutritional support, psychosocial care, and symptom management to create a holistic survivorship care plan. More studies are needed to understand the impact of physical activity on different populations, including older adults, those with comorbidities, and underserved groups. This will help address disparities in access to exercise programs and optimize outcomes across diverse patient populations. Healthcare providers play a crucial role in counseling cancer survivors on the benefits of physical activity and providing appropriate referrals to exercise professionals or programs tailored for cancer survivors. These perspectives highlight the need for ongoing research and integration of physical activity into cancer care to optimize long-term outcomes for survivors. Additionally, it would be valuable to investigate the underlying biological mechanisms through which exercise influences immune cell dynamics, particularly focusing on the role of cytokines and other immunological markers over extended periods. Furthermore, incorporating advanced techniques such as single-cell RNA sequencing could provide deeper insights into the functional states of immune cells and their roles in cancer immunity in response to physical activity.

The findings of this study underscore the potential role of structured exercise interventions as an integral component of comprehensive cancer care. Clinicians should consider implementing personalized exercise programs for patients undergoing chemotherapy to enhance immune function. Given the demonstrated stability or increase in specific immune cell subsets among patients engaged in supervised physical activities, healthcare providers can advocate for these interventions to increase immune system versus tumor response or to at least to some extent mitigate immunosuppressive effects of chemotherapy. Taken together, similar excercise interventions as presented in study potentially improve clinical outcomes and enhances the overall well-being of cancer patients.

B-lymphocytes are gaining recognition for their vital role in combating cancer by contributing to anti-tumor immune responses. Numerous studies indicate that B cells can boost anti-tumor immunity through mechanisms such as antibody production, antigen presentation, and direct interactions with other immune cells [43].

For example, in the context of acute myeloid leukemia (AML), B cells have been shown to influence the tumor microenvironment, enhancing the recruitment and activation of immune cells like T cells and natural killer cells. Similarly, studies on small cell lung cancer (SCLC) metastases have found that tumor-infiltrating lymphocytes (TILs) containing B cells can significantly affect patient outcomes by modulating immune responses to tumor cells and potentially improving the effectiveness of immunotherapy [44,45].

Furthermore, research on hematological cancers suggests that B-lymphocytes are not merely passive participants but actively contribute to the anti-tumor response, particularly when immune checkpoint inhibitors are utilized. B cells can influence the expression of checkpoint molecules like PD-1 or PD-L1 on other immune cells, thereby enhancing the efficacy of these therapies [46].

These insights highlight the potential for developing B cell-based strategies or therapies that enhance B cell function as part of comprehensive cancer immunotherapy regimens. In this regard, a supportive care such as monitored physical activity or training encouragement may provide additional benefit. Further studies are required to fully understand how B cells interact with the tumor microenvironment and contribute to the immune landscape across various cancers.

## 4 Conclusion

There has been enough evidence presented about exercise affecting blood cell counts in healthy adults. Less randomized prospective studies describe effect of chronic exercise on immune cells in cancer patients. In our prospective randomized study, we observed a significant increase of absolute B cell numbers in both exercise (SAPA) and control (CO) group of patients with breast cancer. The rate of increase was significantly higher in SAPA group than in CO group suggesting a possible beneficial effect of exercise on the immune system in cancer patients via this phenomenon. Since B cells have been confirmed to infiltrate tumor microenvironment and various studies confirmed their role in inhibition of tumor proliferation, they may contribute on exercise-related anti-tumor immune-mediated effect. Further studies on a higher number of patients need to be conducted to confirm our results and to elucidate the clinical impact of our findings.

## CRediT authorship contribution statement

**Rihacek Michal:** Validation, Supervision, Methodology, Investigation, Formal analysis, Conceptualization. **Wagnerova Andrea:** Visualization, Resources, Methodology, Investigation, Data curation. **Halamkova Jana:** Writing – original draft, Supervision, Project administration, Methodology, Investigation, Funding acquisition, Data curation. **Pehalova Lucie:** Writing – original draft, Supervision, Formal analysis, Data curation. **Rihackova Eva:** Writing – original draft, Visualization, Validation, Conceptualization. **Boucek Lubos:** Validation, Formal analysis, Data curation. **Kapounková Kateřina:** Supervision, Funding acquisition, Formal analysis, Conceptualization. **Hrnčiříková Iva:** Resources, Methodology, Funding acquisition, Conceptualization. **Kiss Igor:** Writing – original draft, Project administration, Funding acquisition, Data curation.

## Institutional review board statement

The study was conducted according to the guidelines of the Declaration of Helsinki and approved by the Institutional Ethics Committee of the Masaryk Memorial Cancer Institute, protocol code 2020/1513/MOU, date of approval June 16, 2020.

## Informed consent statement

Informed consent was obtained from all subjects involved in this study.

## Data availability statement

The data presented in this study are available on request from the corresponding author.

## Funding

This work was supported by the Ministry of the Health of the Czech Republic, MH CR—DRO (MMCI, 00209805), and grant NU21-09-00558. All rights reserved. This research was also supported by LRI projects CZECRIN (no. LM2023049) and BBMRI-CZ (no. LM2023033).

## Declaration of competing interest

The authors declare that they have no known competing financial interests or personal relationships that could have appeared to influence the work reported in this paper.
